# COVID-19 infection and decline in outdoor activities associated with depression in older adults: A multicenter study in Vietnam

**DOI:** 10.1371/journal.pone.0286367

**Published:** 2023-06-23

**Authors:** Huan Thanh Nguyen, Thien Hoang Le, Chanh Cong Nguyen, Thanh Dinh Le, Tan Van Nguyen

**Affiliations:** 1 Department of Geriatrics and Gerontology, University of Medicine and Pharmacy at Ho Chi Minh city, Ho Chi Minh City, Vietnam; 2 Thong Nhat Hospital, Ho Chi Minh City, Vietnam; 3 Department of Geriatrics and Gerontology, Pham Ngoc Thach University of Medicine, Ho Chi Minh City, Vietnam; Bach Mai Hospital, VIET NAM

## Abstract

**Background:**

The coronavirus disease (COVID-19) pandemic has caused a serious global communicable disease burden. Although COVID-19 and its policy responses have significantly influenced older adults, the impact of COVID-19 on depression in the older population is not fully understood. We aimed to investigate whether a history of COVID-19 infection and a decline in outdoor activities during the COVID-19 pandemic were associated with depression among older adults in Vietnam.

**Methods:**

This multicenter cross-sectional study was conducted on 1,004 outpatients (aged ≥60 years; mean age 70.8 ± 7.3 years; men, 33.0%) visiting three hospitals for a comprehensive geriatric assessment between November 2021 and July 2022. Depression over the past week was evaluated using the 15-item Geriatric Depression Scale. History of COVID-19 infection and decline in outdoor activities were included as binary variables. We adjusted these two factors with sociodemographic and geriatric variables and comorbidities using a logistic regression analysis in separate models.

**Results:**

A total of 156 participants (15.5%) experienced depression. The proportion of mild, moderate, and severe depressive symptoms was 14.1%, 44.9%, and 41.0%, respectively. In the multivariate model, decline in outdoor activities (odds ratio [OR] 17.2, 95% confidence interval [CI] 9.15–32.2, *p* <0.001) and history of COVID-19 infection (OR 2.22, 95% CI 1.28–3.84, *p* = 0.004) were associated with depression. Additionally, we found that age ≥ 75 years, female sex, being underweight, limitations in functional status, poor sleep quality, and stroke were associated with depression. Of the associated factors, decline in outdoor activities had a moderate strength of association with depression (r = 0.419), while each of the remaining factors had a weak strength of association.

**Conclusions:**

COVID-19 had a direct and indirect impact on depression in older adults, reflecting an association between both a history of COVID-19 infection and a decline in outdoor activities during the COVID-19 pandemic and depression in the older population.

## Introduction

The coronavirus disease (COVID-19) pandemic has spread worldwide since December 2019, and the first COVID-19 cases in Vietnam were reported in January 2020 (**S1 Table**) [1]. Although there has been an epidemiological change from communicable diseases to a higher incidence of noncommunicable diseases in Vietnam [2], the infectious outbreak has fueled economic and social upheaval in the country [3]. As of March 2023, Vietnam has experienced four waves of the COVID-19 pandemic, with 11,527,054 confirmed COVID-19 cases (11,660 cases per 100,000 people) and 43,186 COVID-19-related deaths (43.7 deaths per 100,000 people) [3, 4]. As with other countries in the world, Vietnam has effectively implemented several policy measures, including social isolation and launching a large, rapid COVID-19 vaccination campaign for the entire population to control COVID-19 transmission and to reduce the number of COVID-19 deaths [5]. However, COVID-19 has negatively impacted both the physical and mental health of the population [6, 7], as have the drastic policy responses to the pandemic.

The proportion of people aged ≥60 years will be 14.2% and 14.3% of the general population worldwide and in Vietnam, respectively, by 2023 [8]. Over the four waves of the COVID-19 pandemic, Vietnam has reported 6.3% of infected people and 50.4% of deaths due to COVID-19 in those ≥65 years [9]. Older adults in Vietnam were required to follow the same safety measures as the general population and were prioritized in the national COVID-19 vaccination campaign launched in March 2021 [4, 10]. During the pandemic, older adults face a greater risk of COVID-19-related complications and mortality owing to immunosenescence and comorbidities [11]. They are also susceptible to depression induced by COVID-19 [12–16]. The potential mechanisms linking COVID-19 infection to depression in older adults may be related to psychological factors such as fear of death based on a higher rate of COVID-19-related mortality in people ≥60 years compared with the younger age group [17, 18] and pathological factors such as inflammation, mitochondria disorder, and hippocampus disorder induced by COVID-19 [19]. In addition, older adults may experience depression due to barriers to healthcare access, social isolation stress, and less time spent outdoors [19, 20]. Previous studies have shown that older adults who spend less time outdoors may have a higher risk of depression [21, 22], and access to outdoor spaces can improve the mental health of older adults during the COVID-19 pandemic [23]. Therefore, examining decline in outdoor activity as a predictor of depression in older adults is warranted.

Although mental health issues were reported more frequently in younger than older people during the pandemic [24], accumulating data have shown that depression presented in 13.4–40.1% of older people [12–16]. However, in these studies, some geriatric issues, such as frailty and sleep quality, were not evaluated, and the link between COVID-19 infection and decline in outdoor activities and depression in the older population was not fully demonstrated. Therefore, the present study aimed to determine the rate of depression among older outpatients using the 15-item Geriatric Depression Scale (GDS-15) and to investigate whether a history of COVID-19 infection and a decline in outdoor activities during the COVID-19 pandemic were associated with depression in the older population in Vietnam.

## Materials and methods

### Study design, participants, and data collection

This multicenter cross-sectional study was conducted between November 2021 and July 2022 among outpatients ≥60 years at three hospitals in Vietnam: Cho Ray Hospital, Gia Dinh People’s Hospital, and University Medical Center of Ho Chi Minh City. Participants were recruited by non-probability sampling. After providing written informed consent, all participants underwent a comprehensive geriatric assessment [25], including physical, functional, psychological, and social assessment by trained geriatricians. Sociodemographic characteristics and comorbidities were obtained from electronic medical records. Exclusion criteria were severe illness (e.g., acute myocardial infarction), active malignancy, missing data, prior clinical diagnosis of depression based on electronic medical records or failure to complete all 15 items of the GDS, and serious mental illness, including bipolar disorder, schizophrenia, post-traumatic stress disorder, and major depressive disorder. The study was conducted in accordance with the ethical principles stated in the Declaration of Helsinki and was approved by the Ethics Committee of the University of Medicine and Pharmacy at Ho Chi Minh City, Vietnam (reference number: 683/HDDD-DHYD November 24, 2021).

### Assessment of depression and independent variables

We used the short form (15 questions) of the GDS to assess depressive symptoms over 1 week in participants through face-to-face interviews [26]. Participants received one point for answering “no” to questions numbered 1, 5, 7, 11, and 13 and one point for answering “yes” to any of the remaining 10 questions. Participants were diagnosed with depression if their total score was 6 or more. Scores of 6–7, 8–10, and 11–15 indicate mild, moderate, and severe depression, respectively [27].

Variables reflecting the impact of COVID-19: In the present study, having a history of COVID-19 infection and a decline in outdoor activities during the COVID-19 pandemic were used to reflect the direct and indirect impacts of COVID-19, respectively. Participants self-reported their history of COVID-19; only older adults diagnosed with COVID-19 by polymerase chain reaction were included. As depressive disorders over 1 week were evaluated, older adults with a COVID-19 recovery time of less than 1 week were excluded. Decline in outdoor activities during the COVID-19 pandemic was qualitatively assessed using two sequent items. First, participants were asked, “Compared to before the COVID-19 pandemic, have you spent less time on outdoor activities?”. They could choose among the following three response options: (1) “Less than before,” (2) “More than before,” and (3) “No change.” Only those who answered “Less than before” were asked the second question: “Compared to before the COVID-19 pandemic, which outdoor activities have you spent less time on?”. Only those who reported at least one outdoor activity they did less often than before the COVID-19 pandemic were coded as experiencing a decline in outdoor activities. Caregivers may be asked to confirm the information provided by the participants.

Socio-demographic variables: Age as a continuous variable and gender as a binary variable were obtained from electronic medical records. Gender was categorized as men or women. Participants were classified into two groups: ages 60 to 74 years (youngest-old) and age ≥75 years (middle-old and oldest-old). They resided in both urban and rural areas. Educational attainment was stratified as pre-senior high school (below tenth grade), senior high school (from tenth grade to twelfth grade), and tertiary education (college, university, or postgraduate education). Marital status included married, widowed, and single/divorced. Working after retirement age was defined as having any type of unpaid or paid job. Alcohol intake was defined as the consumption of any alcohol in the past 1 month. According to the World Health Organization guidelines for the Asia-Pacific region, body mass index (BMI) was classified as underweight (<18.5 kg/m^2^), normal weight (18.5–22.9 kg/m^2^), overweight (23.0–24.9 kg/m^2^), and obese (≥25 kg/m^2^) [28].

Geriatric characteristics: Polypharmacy was defined as taking five or more medications, and multimorbidity was defined as the presence of two or more chronic diseases [29, 30]. Functional status was evaluated using the Katz activities of daily living (ADLs) and the Lawton instrumental activities of daily living (IADLs) indices [31, 32]. Participants were coded as having limitations in ADLs or IADLs if they self-reported being unable to complete one or more tasks for each index. Frailty was screened using the Program of Research to Integrate Services for the Maintenance of Autonomy 7 (PRISMA-7) questionnaire with seven questions, each scoring 0 or 1. A total score ≥3 was considered indicative of frailty [33]. Sleep quality during the past month was assessed using the self-reported Pittsburgh Sleep Quality Index (PSQI). A total score ≥5 indicated poor sleep quality [34]. As depression over 1 week was evaluated, participants with new‐onset poor sleep quality during the past week were excluded.

### Sample size calculation

We calculated the sample size for estimating the association between main predictors and depression using the following formula for estimating the odds ratio [35]: n = [(r+1)/r)*[p*(1-p)*(Z_1-β_ + Z_1-α/2_)^2^/(p_1_-p_2_)^2^], where n = the required sample size, r = control (without COVID-19 infection) to cases (with COVID-19 infection) ratio (assumed to be 1), Z_1-β_ = desired power (0.84 for 80% power), Z_1- α/2_ = 1.96 (with α = 0.05, and 95% confidence interval), p = proportion of population = (p_1_ + p_2_)/2, p_1_ and p_2_ = expected proportion in cases and control (assumed to be 0.4 and 0.3), respectively. This study required a minimum of 366 participants. We also calculated the sample size to estimate the rate of depression using a single population proportion formula [35]: n = Z^2^_1- α/2_*[p*(1-p)/d^2^], where n = the required sample size, Z^2^_1- α/2_ = 1.96 (with α = 0.05, and 95% confidence interval), and d = precision (assumed to be 0.03). Previous studies have shown that depression ranged from 13.4% to 40.1% of the older population during the COVID-19 pandemic [12–16]. Therefore, 495 to 1,025 participants were required for both study aims.

### Statistical analyses

Categorical variables were described as frequencies and percentages (%). The chi-square test or Fisher’s exact test was used to compare the categorical variables. Continuous variables were described using means and standard deviations. The Student’s t-test was used to determine the statistical significance of the difference between the means of the two study groups. Univariate logistic regression was used to identify the potential factors associated with depression. Multicollinearity was assessed using variance inflation factor. Interaction between two independent variables was detected by the statistical significance of the interaction term of the product of two independent variables. Confounding was identified as a percentage difference of more than 10% between unadjusted and adjusted odds ratios. Multivariate logistic regression analyses were conducted with the two primary predictors (COVID-19 infection and decline in outdoor activities) in separate models. Only variables that were significant at *p* <0.05 in the univariate analysis were included in the multivariate logistic regression. An effect size for each variable was determined by Pearson correlation coefficient (r). The significance level was set at *p* <0.05. The study data were analyzed using IBM SPSS Statistics version 25 (IBM Corp., Armonk, NY, USA).

## Results

**Fig 1:**
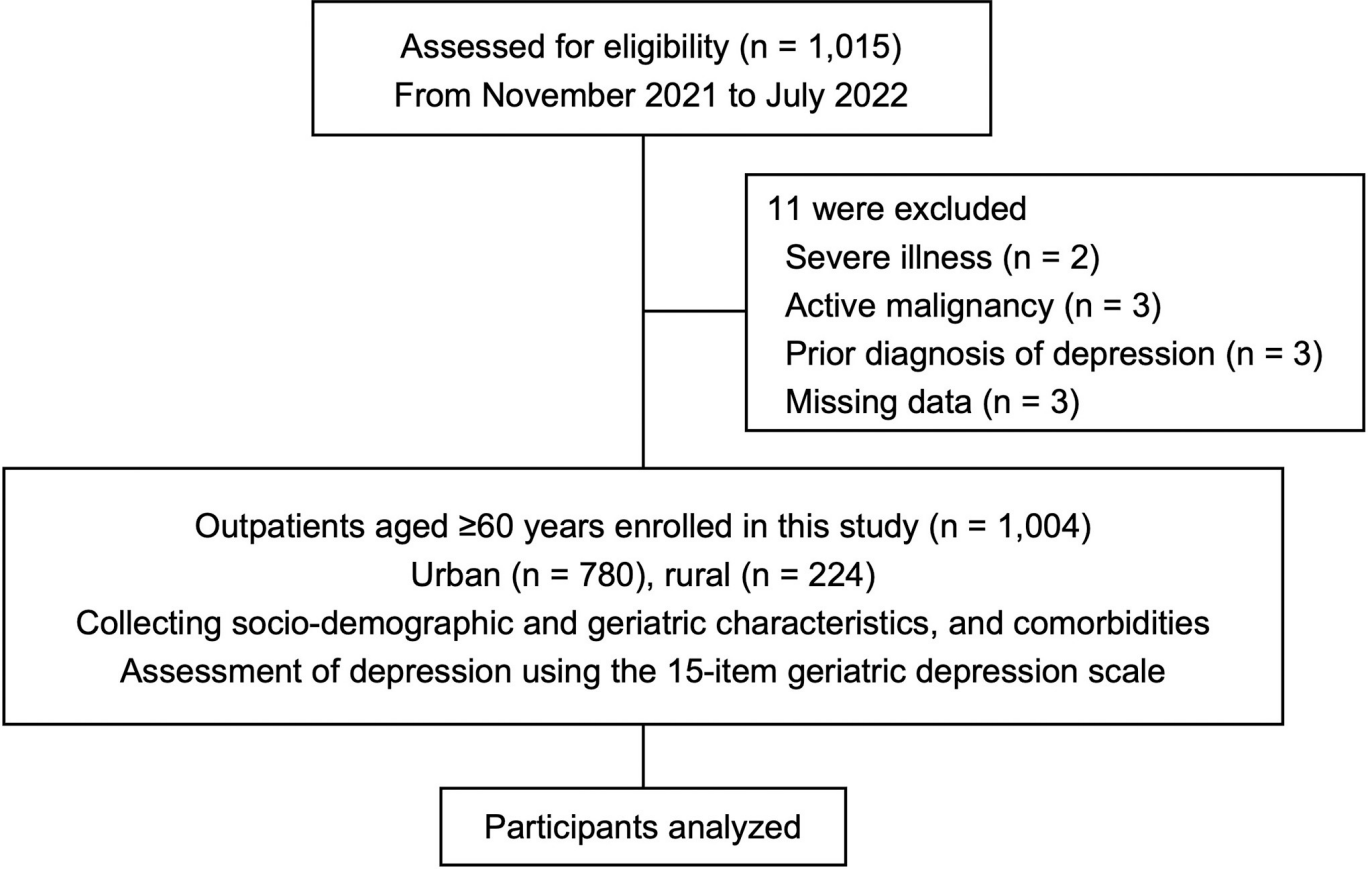
Flow diagram for participant enrollment.

Our study enrolled 1,004 older adults with a mean age of 70.8 ± 7.3 (range, 60–95) years and a female predominance (67.0%). **Fig 1** shows the flow diagram of participant enrollment. We identified 156 older adults (15.5%) who had depression over the past week. Among those with depression, the proportion of mild, moderate, and severe depressive disorders was 14.1%, 44.9%, and 41.0%, respectively. A total of 547 participants (54.5%) reported a history of COVID-19 infection and 429 participants (42.7%) had a decline in outdoor activities. **S2 Table** shows the rates of decline in outdoor activities. **Table 1** shows the baseline characteristics of participants according to their depression status. Regarding socio-demographic characteristics, the depression group was significantly older and had a lower proportion of those working after retirement age; it also had higher proportions of history of COVID-19 infection and decline in outdoor activities than the non-depression group. There were significant differences in educational attainment, marital status, and BMI between the two groups. During the geriatric assessment, older participants with depression had more limitations in ADLs and IADLs, frailty, and poor sleep quality than those without depression. Hypertension was the most common medical condition in the study population, and stroke, osteoarthritis, and chronic kidney disease were reported more frequently in older participants with depression than in those without depression.

**Table 1:** Baseline characteristics of participants according to depression.

Characteristics	Total (n = 1,004)	Depression (n = 156)	Non-depression (n = 848)	*p* value^a^
Socio-demographic characteristics				
Age, years	70.8 ± 7.3	73.5 ± 8.4	70.3 ± 7.0	<0.001
Age ≥ 75 years, n (%)	281 (28.0)	64 (41.0)	217 (25.6)	<0.001
Gender, n (%)				<0.001
Men	331 (33.0)	28 (17.9)	303 (35.7)	
Women	673 (67.0)	128 (82.1)	545 (64.3)	
Living region				0.069
Urban	780 (77.7)	112 (71.8)	668 (78.8)	
Rural	224 (22.3)	44 (28.2)	180 (21.2)	
Level of education, n (%)				<0.001
Pre-senior high school	634 (63.2)	120 (76.9)	514 (60.6)	
Senior high school	235 (23.4)	29 (18.6)	206 (24.3)	
Tertiary education	135 (13.4)	7 (4.5)	128 (15.1)	
Marital status, n (%)				0.006
Married	711 (70.8)	97 (62.2)	614 (72.4)	
Widowed	218 (21.7)	49 (31.4)	169 (19.9)	
Single or divorced	75 (7.5)	10 (6.4)	65 (7.7)	
Working after retirement age, n (%)	111 (11.1)	8 (5.1)	103 (12.1)	0.008
Alcohol intake, n (%)	43 (4.3)	5 (3.2)	38 (4.5)	0.666
History of COVID-19 infection, n (%)	547 (54.5)	105 (67.3)	442 (52.1)	<0.001
Decline in outdoor activities, n (%)	429 (42.7)	142 (91.0)	287 (33.8)	<0.001
BMI, kg/m^2^	22.5 ± 3.0	21.4 ± 3.7	22.7 ± 2.9	<0.001
BMI groups, n (%)				<0.001
Underweight	510 (50.8)	70 (44.9)	440 (51.9)	
Normal	96 (9.6)	41 (26.2)	55 (6.5)	
Overweight	210 (20.9)	21 (13.5)	189 (22.3)	
Obese	188 (18.7)	24 (15.4)	164 (19.3)	
Geriatric characteristics				
Polypharmacy, n (%)	443 (44.1)	62 (39.7)	381 (44.9)	0.254
Multimorbidity, n (%)	731 (72.8)	113 (72.4)	618 (72.9)	0.922
Limitations in ADLs, n (%)	46 (4.6)	26 (16.7)	20 (2.4)	<0.001
Limitations in IADLs, n (%)	252 (25.1)	92 (59.0)	160 (18.9)	<0.001
Frailty, n (%)	245 (24.4)	89 (57.1)	156 (18.4)	<0.001
Poor sleep quality, n (%)	673 (67.0)	148 (94.9)	525 (61.9)	<0.001
Medical history, n (%)				
Hypertension	818 (81.5)	131 (84.0)	687 (81.0)	0.433
Coronary artery disease	274 (27.3)	33 (21.2)	241 (28.4)	0.064
Diabetes mellitus	235 (23.4)	45 (28.8)	190 (22.4)	0.099
Stroke	52 (5.2)	24 (15.4)	28 (3.3)	<0.001
Osteoarthritis	401 (39.9)	79 (50.6)	322 (38.0)	0.003
Chronic pulmonary diseases	53 (5.3)	10 (6.4)	43 (5.1)	0.442
Chronic venous insufficiency	154 (15.3)	30 (19.2)	124 (14.6)	0.147
Chronic kidney disease	197 (19.6)	42 (26.9)	155 (18.3)	0.016

**Note:** ^a^depression group vs non-depression group

**Abbreviations:** ADLs, activities of daily living; IADLs, instrumental activities of daily living; BMI, body mass index.

**Table 2** shows characteristics of the depressive disorders evaluated using the GDS-15. In our study, the five questions with “no” answers indicating depression had the lowest proportions. The question with the highest proportion of “yes” answers indicating depression was “Do you think that most people are better off than you are?”, followed by the questions “Do you prefer to stay at home, rather than going out and doing things?” and “Are you afraid that something bad will happen to you?”.

**Table 2:** Prevalence of item endorsement on the 15-item GDS in 156 participants with depression.

	Questions	Answers indicate depression, n (%)	
1	Are you basically satisfied with your life?	No	66 (42.3)
2	Have you dropped many of your activities and interests?	Yes	131 (84.0)
3	Do you feel that your life is empty?	Yes	101 (64.7)
4	Do you often get bored?	Yes	135 (86.5)
5	Are you in good spirits most of the time?	No	66 (42.3)
6	Are you afraid that something bad is going to happen to you?	Yes	137 (87.8)
7	Do you feel happy most of the time?	No	65 (41.7)
8	Do you often feel helpless?	Yes	115 (73.7)
9	Do you prefer to stay at home, rather than going out and doing things?	Yes	141 (90.4)
10	Do you feel that you have more problems with memory than most?	Yes	132 (84.6)
11	Do you think it is wonderful to be alive now?	No	74 (47.4)
12	Do you feel worthless the way you are now?	Yes	96 (61.5)
13	Do you feel full of energy?	No	63 (40.4)
14	Do you feel that your situation is hopeless?	Yes	92 (59.0)
15	Do you think that most people are better off than you are?	Yes	144 (92.3)

**Note:** Participants received one point when answering “no” to questions numbered 1, 5, 7, 11, and 13, and one point when answering “yes” to any of the remaining 10 questions.

**Table 3** shows the factors associated with depression in the univariate regression. **S3–S5 Tables** show the assessment of multicollinearity, interaction, and confounding, respectively. We made decisions about the models based on the -2 log likelihood statistic (**S6 Table**). In the two separate models of multivariate regression analyses for the two primary predictors, we found that both a history of COVID-19 infection and a decline in outdoor activities were associated with depression in older adults. In addition, age ≥ 75 years, female sex, being underweight, limitations in functional status, poor sleep quality, and stroke were associated with depression in the two multivariate models. Decline in outdoor activities had a moderate strength of association with depression (r = 0.419), while the remaining factors had weak strengths of association (**Table 4**).

**Table 3:** Factors associated with depression in the univariate regression (n = 1,004).

Variables	Unadjusted OR (95% CI)	*p* value
Age ≥ 75 years	2.02 (1.42–2.88)	<0.001
Women	2.54 (1.65–3.92)	<0.001
Rural	1.32 (0.89–1.96)	0.162
Working after retirement age	0.39 (0.19–0.82)	0.013
Marital status		
Married	1	
Widowed	1.84 (1.25–2.69)	0.002
Single or divorced	0.97 (0.48–1.96)	0.941
Level of education		
Pre-senior high school	1	
Senior high school	0.63 (0.39–0.93)	0.023
Tertiary education	0.23 (0.11–0.51)	<0.001
BMI group		
Normal	1	
Underweight	4.69 (2.90–7.55)	<0.001
Overweight	0.70 (0.42–1.17)	0.173
Obese	0.92 (0.56–1.51)	0.742
Multimorbidity	0.98 (0.67–1.43)	0.909
Polypharmacy	0.69 (0.49–0.98)	0.039
Decline in outdoor activities	19.8 (11.2–34.9)	<0.001
Limitations in ADLs	8.28 (4.49–15.3)	<0.001
Limitations in lADLs	6.18 (4.30–8.88)	<0.001
Frailty	5.89 (4.11–8.46)	<0.001
Poor sleep quality	12.0 (7.29–19.8)	<0.001
History of COVID-19 infection	1.89 (1.32–2.71)	0.001
Alcohol intake	0.71 (0.27–1.82)	0.472
Hypertension	1.23 (0.78–1.95)	0.382
Coronary artery disease	0.68 (0.45–1.02)	0.062
Diabetes mellitus	1.40 (0.96–2.06)	0.082
Stroke	5.33 (2.30–9.47)	<0.001
Osteoarthritis	1.68 (1.19–2.36)	0.003
Chronic pulmonary diseases	1.99 (1.08–3.68)	0.028
Chronic venous insufficiency	1.39 (0.89–2.16)	0.144
Chronic kidney disease	1.65 (1.11–2.44)	0.013

**Abbreviations:** ADLs, activities of daily living; BMI, body mass index; CI, confidence interval; IADLs, instrumental activities of daily living; OR, odds ratio.

**Table 4:** Factors associated with depression in the multiple regression analysis (n = 1,004).

Variables	Model 1		Model 2		
	Adjusted OR (95% CI)	*p* value	Adjusted OR (95% CI)	*p* value	Effect size (r)
Age ≥75 years	2.17 (1.16–4.06)	0.015	2.61 (1.37–4.98)	0.004	0.125
Women	2.58 (1.49–4.47)	0.001	3.41 (1.86–6.25)	<0.001	0.137
Working after retirement age					
Marital status					
Married					
Widowed					
Single or divorced					
Level of education					
Pre-senior high school					
Senior high school					
Tertiary education					
BMI group					0.029
Normal	1		1		
Underweight	3.05 (1.69–5.52)	<0.001	2.94 (1.54–5.60)	0.001	
Overweight					
Obese					
Polypharmacy					
Frailty					
Limitations in ADLs	4.09 (1.89–8.82)	<0.001	4.70 (2.01–10.9)	<0.001	0.248
Limitations in lADLs	3.17 (1.49–6.78)	0.003	3.09 (1.38–6.93)	0.006	0.335
Poor sleep quality	6.65 (3.03–14.6)	<0.001	5.38 (2.37–12.2)	<0.001	0.254
Decline in outdoor activities	N/A		17.2 (9.15–32.2)	<0.001	0.419
History of COVID-19 infection	2.22 (1.28–3.84)	0.004	N/A		0.110
Stroke	2.94 (1.42–6.06)	0.004	3.42 (1.50–7.78)	0.003	0.198
Osteoarthritis	1.57 (1.03–2.41)	0.037			0.094
Chronic pulmonary diseases					
Chronic kidney disease					

**Note:** Multivariate logistic regression analyses were conducted with the two primary predictors in two separate models: Model 1 for history of COVID-19 infection and Model 2 for decline in outdoor activities. Only variables with *p* values <0.05 in the univariate analysis were selected for multivariate logistic regression. Only variables with a *p* value <0.05 in the multiple regression are shown. An effect size for each variable was determined by Pearson correlation coefficient (r).

**Abbreviations:** BMI, body mass index; CI, confidence interval; N/A, not applicable; OR, odds ratio.

## Discussion

The COVID-19 pandemic has had considerable global mental health impacts and affected the psychological conditions of the older population [12–16, 24]. However, evidence on the impact of COVID-19 on depression among older adults remains limited. We found that 15.5% of older outpatients in Vietnam had experienced depression over the past week. Our study also demonstrated that a history of COVID-19 infection and a decline in outdoor activities were the two COVID-19-related factors associated with depression in the older population. Based on the main findings, we propose the following two points for discussion.

### Depression in older adults

A systematic literature review showed an increase in the prevalence of depressive and anxiety disorders in most countries worldwide during the COVID-19 pandemic as compared to before the pandemic [24]. The increased burden of psychological disorders may be due to the fear of COVID-19, forced adaptation of novel daily behaviors to aggressive measures, barriers to social support and healthcare access, limited contact, feelings of loneliness, loss of income, and decreased physical outdoor activities during the pandemic [6, 7, 12, 36, 37]. However, the impact of COVID-19 on mental health has varied across countries. In the general population, a previous study showed that among middle-income countries in Asia, Vietnam had the lowest rates of mental disorders during the COVID-19 pandemic, which can be explained by differences in socio-demographic, economic, cultural, and lifestyle factors [7]. Furthermore, Vietnam’s effective policy responses to the pandemic may have also contributed to citizens’ satisfaction [5, 38].

Some studies have shown a substantial burden of depression among older adults during the COVID-19 pandemic, with different rates depending on the studied population and methods used to define depression [15, 16, 39]. In the older Vietnamese population, two previous studies conducted before the COVID-19 pandemic showed high rates of depression among older adults living in rural (26.4%) and urban (66.9%) areas [40, 41]. Interestingly, during the COVID-19 pandemic, lower rates of depression among older adults were found in our study and that by Do *et al*. (13.4%) [12]. The following three factors may have contributed to these findings. First, while Do *et al*. [12] conducted their study at the beginning of the pandemic with no restrictions, the present study recruited participants at the end of the fourth COVID-19 wave and the beginning of loosening of restrictions. This relaxation of anti-COVID-19 measures may have provided opportunities for older adults to engage in outdoor activities and influenced the burden of depression. However, because of lack of studies performed in Vietnam during periods of strict measures, speculations regarding the effect of relaxation measures on depression must be confirmed by further research. Second, living arrangements may have influenced depression among the older population [42]. Unlike older people in developed countries who spend their later lives living with no adult children or in long-term care facilities [43], the majority of Vietnamese older adults are cared for by their offspring in houses where many generations live together [44]. This notion is supported by previous literature indicating that family relationships play an important role in shaping the well-being and mental health of older adults [45]. In addition, during social isolation, most family members stayed at home and were likely to have more time for positive family relationships, and younger members could provide more assistance to older ones. Third, since income loss has been associated with psychological disorders, young people are more likely to become unemployed during the COVID-19 pandemic than older people [46]. In Vietnam, according to the Longitudinal Study of Aging and Health, older adults received their main source of income from retirement pensions, social welfare benefits, and support from their adult children or grandchildren [47]. Further studies are needed to clarify how diverse family structures and intergenerational relationships, as well as income, can influence the psychological conditions of older adults during COVID-19 and other pandemics.

Our study found that limitations in functional status, frailty, poor sleep quality, and certain chronic diseases, including stroke, osteoarthritis, and chronic kidney disease, were more significantly prevalent in the depression group than the non-depression group. Since geriatric issues and comorbidities can result in diminished life expectancy and quality of life in older adults [48–51], these medical issues should be evaluated in clinical practice.

### Factors associated with depression

The development of depressive disorders can be related to molecular mechanisms or the social environment of individuals with certain risk factors [52, 53]. Since depression is potentially preventable, early recognition of factors that influence psychological conditions is necessary to identify older adults with a high risk of depression who may require further appropriate assessment and management [54]. Several studies have revealed many factors associated with depression among older adults both before [40, 41, 55] and during the COVID-19 pandemic [12, 13, 15, 24]. Our results demonstrated that a history of COVID-19 and a decline in outdoor activities were associated with depression in older adults during the pandemic.

To the best of our knowledge, no study has explored the direct relationship between COVID-19 and depressive disorders among older adults. The results of our analysis are in accordance with those of subgroup analyses in a previous study suggesting that COVID-19 infection is associated with depressive symptoms in older adults [56]. A previous study found no association between COVID-19 and depressive symptoms in young individuals [56]. These previous findings, together with ours, suggest that although depressive and anxiety disorders occurred more frequently in younger people than in older people during the pandemic [24], older adults may be more vulnerable to depression than younger adults after COVID-19 infection. Since previous evidence indicated that older age is a risk factor for poorer COVID-19 outcomes, such as mortality and complications [57], older individuals may have considerable concern for their health after COVID-19 infection. Furthermore, poorly controlled comorbidities and limited healthcare access during the pandemic may also contribute to depression among older adults. In agreement with this notion, our study found that most participants with depression were afraid that something bad would happen to them.

Previous studies have linked the benefits of routine outdoor activities to physical function and psychosocial outcomes in older adults and found that those who spend more time outdoors tend to have a lower risk of depression [21, 22]. Access to parks, outdoor spaces, and nature can promote the mental health and well-being of older adults during COVID-19 [23]. During the COVID-19 pandemic, although the strategies of social isolation and stay-at-home orders could reduce the risk of virus transmission, strict measures compelled older individuals to make unpredictable, forced changes in their daily life habits, reduced social participation, and limited access to medical services [13–15]. The current study was performed just when COVID-19-related restrictions were being loosened in Vietnam and the Omicron variant was spreading (**S1 Table**). Hence, the decline in outdoor activities of participants in our study is unlikely due to social isolation measures, instead, it may be related to the habit of staying at home after an extended period of strict lockdown or the fear of contracting COVID-19 from public areas. Restrictions on leaving home reduced the time older adults spent outdoors and therefore partially contributed as a factor associated with psychosocial disorders in our study of the older population. However, because of the differences in the levels of policy responses due to COVID-19 across countries and the psychological adaptation of people over time to a pandemic, longitudinal studies are required to better understand the burden of depression among older adults in each wave of the COVID-19 pandemic.

Our study also identified age ≥ 75 years, female sex, being underweight, limitations in functional status, poor sleep quality, and stroke as significantly influencing the extent to which depression increased during the pandemic. First, older age was associated with depression in a recent study; the likelihood of depression was higher in the middle-old than the youngest-old [58]. Second, while women were more affected by depression during the COVID-19 pandemic than men in the general population [24], some studies showed that sex is not a factor for depression in older adults due to the pandemic [13, 15, 16]. However, our study, consistent with that of older Vietnamese adults before the pandemic [55], showed that being a woman was associated with depression in the older population. These findings reflect the characteristics of Vietnamese geographic culture and family structure in that older women play a main role in housekeeping and taking care of their families [47]. They might experience more stress during the COVID-19 lockdown periods when family members stay at home. Third, the negative association of being underweight and depression in our older population is supported by a prior study demonstrating that underweight may develop depressive symptoms in older Asian populations [59]. The reciprocal association remains unclear but may be explained by some biological factors such as lower levels of high density lipoprotein cholesterol (HDL-c) found in individuals who are underweight and those with lower levels of HDL-c being more likely to have depressive symptoms [60]. Fourth, the association of limitations in functional status with depression in our sample may be explained by a study showing that difficulty in self-care activities and tasks of household management may gradually induce an accumulation of depressive symptoms in older adults [61]. Fifth, sleep disturbance and depression were frequently found during the first wave of the COVID-19 pandemic [62]. These two health issues have a well-known bidirectional relationship in which poor sleep quality presents as both an expression and a risk factor for depression [63]. Sixth, the evidence of post-stroke depression is very clear, based on a meta-analysis indicating that depressive symptoms can occur in approximately one-third of stroke survivors [64]. Taken together, our study found that depression in older adults was associated with COVID-19 and some sociodemographic and geriatric factors, as well as comorbidity. However, although our study found possible influences of some factors on depression, we did not control for all potential confounders, and further studies of the association are needed.

Our study has several limitations. First, there was a lack of information on the severity of COVID-19 infection and post-COVID-19 symptoms in the study population. Furthermore, time elapsed since COVID-19 diagnosis was not collected; thus, the association between COVID-19 infection and depression may be estimated over a relatively short time frame. Second, we only collected some sociodemographic characteristics; thus, the impact of other characteristics, such as retirement income, on depression could not be identified. Third, since we only recruited older adults enrolled in clinics by non-probability sampling, our participants are representative of only older outpatients and not of community-dwelling older adults or hospitalized older patients; thus, our observed results are not generalizable to the older population in Vietnam. Fourth, based on the nature of GDS-15, only depression over the past week was evaluated. We could thus not address prior depression before the pandemic as well as for more than 1 week; therefore, our results could not reflect the entire burden of depression during the COVID-19 pandemic. Finally, the causal relationship between depression and associated factors, such as COVID-19 infection and decline in outdoor activities, could not be evaluated because of the cross-sectional nature of the study design. To clarify the exact relationships, further longitudinal studies are warranted.

## Conclusions

This is the first study to demonstrate that COVID-19 can both directly and indirectly affect depressive disorders among older adults. Both a history of COVID-19 infection and a decline in outdoor activities during the COVID-19 pandemic were associated with depression in the older population. Since the pandemic is ongoing and early detection of depression is important to initiate appropriate intervention, older adults with a history of COVID-19 infection and a decline in outdoor activities should be evaluated for depression in clinical practice.

## Supporting information

S1 Table Timeline of the COVID-19 pandemic in Vietnam. https://doi.org/10.6084/m9.figshare.23522976.v1.
(TXT)

S2 Table Types of declined outdoor activities (n = 429). https://doi.org/10.6084/m9.figshare.23523045.v1.
(TXT)

S3 Table Assessment of multicollinearity for variables with p values <0.05 in the univariate analysis. https://doi.org/10.6084/m9.figshare.23523048.v1.
(TXT)

S4 Table Assessment of interaction between variables with p values <0.05 in the univariate analysis and the two primary predictors. https://doi.org/10.6084/m9.figshare.23523054.v1.
(TXT)

S5 Table Assessment of confounding of the two primary predictors on variables with p values <0.05 in the univariate analysis. https://doi.org/10.6084/m9.figshare.23523069.v1.
(TXT)

S6 Table The -2 log likelihood values of the adjusted models. https://doi.org/10.6084/m9.figshare.23523078.v1.
(TXT)

S1 Dataset Deidentified dataset. https://doi.org/10.6084/m9.figshare.23523087.v1.
(TXT)

## References

[1] Phan LT, Nguyen TV, Luong QC, Nguyen TV, Nguyen HT, Le HQ, et al. Importation and Human-to-Human Transmission of a Novel Coronavirus in Vietnam. N Engl J Med. 2020;382(9):872–4. doi: 10.1056/NEJMc2001272 31991079 PMC7121428

[2] Supakul S, Park HY, Nguyen BN, Giang KB. Prevalence differences in major non- communicable diseases in a low- middle income country: a comparative study between an urban and a rural district in Vietnam. J Glob Health Sci. 2019;1(2):e47. doi: 10.35500/jghs.2019.1.e47

[3] Minh LHN, Khoi Quan N, Le TN, Khanh PNQ, Huy NT. COVID-19 Timeline of Vietnam: Important Milestones Through Four Waves of the Pandemic and Lesson Learned. Front Public Health. 2021;9:709067. doi: 10.3389/fpubh.2021.709067 34900885 PMC8651614

[4] The World Health Organization. Available online at: https://covid19.who.int/region/wpro/country/vn (accessed March 16, 2023).

[5] Le TT, Vodden K, Wu J, Atiwesh G. Policy Responses to the COVID-19 Pandemic in Vietnam. Int J Environ Res Public Health. 2021;18(2). doi: 10.3390/ijerph18020559 33440841 PMC7828055

[6] Shaukat N, Ali DM, Razzak J. Physical and mental health impacts of COVID-19 on healthcare workers: a scoping review. Int J Emerg Med. 2020;13(1):40. doi: 10.1186/s12245-020-00299-5 32689925 PMC7370263

[7] Wang C, Tee M, Roy AE, Fardin MA, Srichokchatchawan W, Habib HA, et al. The impact of COVID-19 pandemic on physical and mental health of Asians: A study of seven middle-income countries in Asia. PLoS One. 2021;16(2):e0246824. doi: 10.1371/journal.pone.0246824 33571297 PMC7877638

[8] Population Pyramids of the World from 1950 to 2100. Available online at: https://www.populationpyramid.net (accessed March 16, 2023).

[9] Ministry of Health Potal. Available online at: https://covid19.gov.vn/ (accessed September 23, 2021).

[10] Tung LT. Social Responses for Older People in COVID-19 Pandemic: Experience from Vietnam. J Gerontol Soc Work. 2020;63(6–7):682–7. doi: 10.1080/01634372.2020.1773596 32501146

[11] Nikolich-Zugich J, Knox KS, Rios CT, Natt B, Bhattacharya D, Fain MJ. SARS-CoV-2 and COVID-19 in older adults: what we may expect regarding pathogenesis, immune responses, and outcomes. Geroscience. 2020;42(2):505–14. doi: 10.1007/s11357-020-00186-0 32274617 PMC7145538

[12] Do BN, Nguyen PA, Pham KM, Nguyen HC, Nguyen MH, Tran CQ, et al. Determinants of Health Literacy and Its Associations With Health-Related Behaviors, Depression Among the Older People With and Without Suspected COVID-19 Symptoms: A Multi-Institutional Study. Front Public Health. 2020;8:581746. doi: 10.3389/fpubh.2020.581746 33313037 PMC7703185

[13] de Oliveira Andrade N, Correia Silva Azambuja H, Carvalho Reis Martins T, Manoel Seixas RA, Moretti Luchesi B. Factors associated with depressive and anxiety symptoms in older adults during the COVID-19 pandemic: a Brazilian study. Aging Ment Health. 2022;26(8):1564–71. doi: 10.1080/13607863.2021.1942431 34225507

[14] Garcia-Portilla P, de la Fuente Tomas L, Bobes-Bascaran T, Jimenez Trevino L, Zurron Madera P, Suarez Alvarez M, et al. Are older adults also at higher psychological risk from COVID-19? Aging Ment Health. 2021;25(7):1297–304. doi: 10.1080/13607863.2020.1805723 32870024

[15] Mistry SK, Ali A, Hossain MB, Yadav UN, Ghimire S, Rahman MA, et al. Exploring depressive symptoms and its associates among Bangladeshi older adults amid COVID-19 pandemic: findings from a cross-sectional study. Soc Psychiatry Psychiatr Epidemiol. 2021;56(8):1487–97. doi: 10.1007/s00127-021-02052-6 33661353 PMC7930102

[16] Kurniawidjaja M, Susilowati IH, Erwandi D, Kadir A, Hasiholan BP, Al Ghiffari R. Identification of Depression Among Elderly During COVID-19. J Prim Care Community Health. 2022;13:21501319221085380. doi: 10.1177/21501319221085380 35333667 PMC8958696

[17] Ozguc S, Kaplan Serin E, Tanriverdi D. Death Anxiety Associated With Coronavirus (COVID-19) Disease: A Systematic Review and Meta-Analysis. Omega (Westport). 2021:302228211050503. doi: 10.1177/00302228211050503 34622711 PMC10768329

[18] Bonanad C, Garcia-Blas S, Tarazona-Santabalbina F, Sanchis J, Bertomeu-Gonzalez V, Facila L, et al. The Effect of Age on Mortality in Patients With COVID-19: A Meta-Analysis With 611,583 Subjects. J Am Med Dir Assoc. 2020;21(7):915–8. doi: 10.1016/j.jamda.2020.05.045 32674819 PMC7247470

[19] Mohammadkhanizadeh A, Nikbakht F. Investigating the potential mechanisms of depression induced-by COVID-19 infection in patients. J Clin Neurosci. 2021;91:283–7. doi: 10.1016/j.jocn.2021.07.023 34373041 PMC8289699

[20] Young DR, Hong BD, Lo T, Inzhakova G, Cohen DA, Sidell MA. The longitudinal associations of physical activity, time spent outdoors in nature and symptoms of depression and anxiety during COVID-19 quarantine and social distancing in the United States. Prev Med. 2022;154:106863. doi: 10.1016/j.ypmed.2021.106863 34774881 PMC8717103

[21] Harada K, Lee S, Lee S, Bae S, Harada K, Suzuki T, et al. Objectively-measured outdoor time and physical and psychological function among older adults. Geriatr Gerontol Int. 2017;17(10):1455–62. doi: 10.1111/ggi.12895 27633728

[22] Jacobs JM, Cohen A, Hammerman-Rozenberg R, Azoulay D, Maaravi Y, Stessman J. Going outdoors daily predicts long-term functional and health benefits among ambulatory older people. J Aging Health. 2008;20(3):259–72. doi: 10.1177/0898264308315427 18332184

[23] Bustamante G, Guzman V, Kobayashi LC, Finlay J. Mental health and well-being in times of COVID-19: A mixed-methods study of the role of neighborhood parks, outdoor spaces, and nature among US older adults. Health Place. 2022;76:102813. doi: 10.1016/j.healthplace.2022.102813 35623164 PMC9127349

[24] Collaborators C-MD. Global prevalence and burden of depressive and anxiety disorders in 204 countries and territories in 2020 due to the COVID-19 pandemic. Lancet. 2021;398(10312):1700–12. doi: 10.1016/S0140-6736(21)02143-7 34634250 PMC8500697

[25] Ellis G, Whitehead MA, Robinson D, O’Neill D, Langhorne P. Comprehensive geriatric assessment for older adults admitted to hospital: meta-analysis of randomised controlled trials. BMJ. 2011;343:d6553. doi: 10.1136/bmj.d6553 22034146 PMC3203013

[26] Sheikh JI, Yesavage JA. Geriatric Depression Scale (GDS): Recent evidence and development of a shorter version. Clinical Gerontologist: The Journal of Aging and Mental Health. 1986;5:165–73. doi: 10.1300/J018v05n01_09

[27] Whooley MA, Browner WS. Association between depressive symptoms and mortality in older women. Study of Osteoporotic Fractures Research Group. Arch Intern Med. 1998;158(19):2129–35. doi: 10.1001/archinte.158.19.2129 9801180

[28] Region WHOWP. The Asia-Pacific perspective: redefining obesity and its treatment. World Health Organization. 2000.

[29] Palmer K, Villani ER, Vetrano DL, Cherubini A, Cruz-Jentoft AJ, Curtin D, et al. Association of polypharmacy and hyperpolypharmacy with frailty states: a systematic review and meta-analysis. Eur Geriatr Med. 2019;10(1):9–36. doi: 10.1007/s41999-018-0124-5 32720270

[30] Vetrano DL, Palmer K, Marengoni A, Marzetti E, Lattanzio F, Roller-Wirnsberger R, et al. Frailty and Multimorbidity: A Systematic Review and Meta-analysis. J Gerontol A Biol Sci Med Sci. 2019;74(5):659–66. doi: 10.1093/gerona/gly110 29726918

[31] Shelkey M, Wallace M. Katz Index of Independence in Activities of Daily Living. J Gerontol Nurs. 1999;25(3):8–9. doi: 10.3928/0098-9134-19990301-05 10362969

[32] Graf C. The Lawton instrumental activities of daily living scale. Am J Nurs. 2008;108(4):52–62; quiz -3. doi: 10.1097/01.NAJ.0000314810.46029.74 18367931

[33] Hebert R, Durand PJ, Dubuc N, Tourigny A, Group P. PRISMA: a new model of integrated service delivery for the frail older people in Canada. Int J Integr Care. 2003;3:e08. doi: 10.5334/ijic.73 16896376 PMC1483944

[34] Smyth C. The Pittsburgh Sleep Quality Index (PSQI). Insight. 2000;25(3):97–8. doi: 10.1067/min.2000.107649 11907900

[35] Charan J, Biswas T. How to calculate sample size for different study designs in medical research? Indian J Psychol Med. 2013;35(2):121–6. doi: 10.4103/0253-7176.116232 24049221 PMC3775042

[36] Le XTT, Dang AK, Toweh J, Nguyen QN, Le HT, Do TTT, et al. Evaluating the Psychological Impacts Related to COVID-19 of Vietnamese People Under the First Nationwide Partial Lockdown in Vietnam. Front Psychiatry. 2020;11:824. doi: 10.3389/fpsyt.2020.00824 32982807 PMC7492529

[37] Yamada M, Kimura Y, Ishiyama D, Otobe Y, Suzuki M, Koyama S, et al. Effect of the COVID-19 Epidemic on Physical Activity in Community-Dwelling Older Adults in Japan: A Cross-Sectional Online Survey. J Nutr Health Aging. 2020;24(9):948–50. doi: 10.1007/s12603-020-1424-2 33155619 PMC7597428

[38] Ivic S. Vietnam’s Response to the COVID-19 Outbreak. Asian Bioeth Rev. 2020;12(3):341–7. doi: 10.1007/s41649-020-00134-2 32837558 PMC7348105

[39] Liang W, Duan Y, Shang B, Hu C, Baker JS, Lin Z, et al. Precautionary Behavior and Depression in Older Adults during the COVID-19 Pandemic: An Online Cross-Sectional Study in Hubei, China. Int J Environ Res Public Health. 2021;18(4). doi: 10.3390/ijerph18041853 33672885 PMC7918441

[40] Dao ATM, Nguyen VT, Nguyen HV, Nguyen LTK. Factors Associated with Depression among the Elderly Living in Urban Vietnam. Biomed Res Int. 2018;2018:2370284. doi: 10.1155/2018/2370284 30596085 PMC6286754

[41] Vu HTT, Lin V, Pham T, Pham TL, Nguyen AT, Nguyen HT, et al. Determining Risk for Depression among Older People Residing in Vietnamese Rural Settings. Int J Environ Res Public Health. 2019;16(15). doi: 10.3390/ijerph16152654 31349566 PMC6696606

[42] Pei Z, Hu F, Qin W, Zhao Y, Zhang X, Cong X, et al. The relationship between living arrangements and depression among older adults in Shandong, China: The mediating role of social support. Front Psychiatry. 2022;13:896938. doi: 10.3389/fpsyt.2022.896938 36451767 PMC9701745

[43] Grabowski DC. The future of long-term care requires investment in both facility- and home-based services. Nature Aging. 2021;1:10–1. doi: 10.1038/s43587-020-00018-y 37117999

[44] Truong SA, Bui TC, Goodkind D, Knodel J. Living arrangements, patrilineality and sources of support among elderly Vietnamese. Asia Pac Popul J. 1997;12(4):69–88. 12293568

[45] Thomas PA, Liu H, Umberson D. Family Relationships and Well-Being. Innov Aging. 2017;1(3):igx025. doi: 10.1093/geroni/igx025 29795792 PMC5954612

[46] Ruengorn C, Awiphan R, Wongpakaran N, Wongpakaran T, Nochaiwong S, Health O, et al. Association of job loss, income loss, and financial burden with adverse mental health outcomes during coronavirus disease 2019 pandemic in Thailand: A nationwide cross-sectional study. Depress Anxiety. 2021;38(6):648–60. doi: 10.1002/da.23155 33793028 PMC8251094

[47] Vu NCT M.T.; Dang L.T.; Chei C.L.; Saito Y. (Eds.). Ageing and Health in Viet Nam. Economic Research Institute for ASEAN and East Asia (ERIA): Jakarta, Indonesia. 2020.

[48] Keeler E, Guralnik JM, Tian H, Wallace RB, Reuben DB. The impact of functional status on life expectancy in older persons. J Gerontol A Biol Sci Med Sci. 2010;65(7):727–33. doi: 10.1093/gerona/glq029 20363833 PMC2884085

[49] Fried LP, Tangen CM, Walston J, Newman AB, Hirsch C, Gottdiener J, et al. Frailty in older adults: evidence for a phenotype. J Gerontol A Biol Sci Med Sci. 2001;56(3):M146–56. doi: 10.1093/gerona/56.3.m146 11253156

[50] Lee M, Choh AC, Demerath EW, Knutson KL, Duren DL, Sherwood RJ, et al. Sleep disturbance in relation to health-related quality of life in adults: the Fels Longitudinal Study. J Nutr Health Aging. 2009;13(6):576–83. doi: 10.1007/s12603-009-0110-1 19536428 PMC3988690

[51] Schoenborn NL, Blackford AL, Joshu CE, Boyd CM, Varadhan R. Life expectancy estimates based on comorbidities and frailty to inform preventive care. J Am Geriatr Soc. 2022;70(1):99–109. doi: 10.1111/jgs.17468 34536287 PMC8742754

[52] Fox ME, Lobo MK. The molecular and cellular mechanisms of depression: a focus on reward circuitry. Mol Psychiatry. 2019;24(12):1798–815. doi: 10.1038/s41380-019-0415-3 30967681 PMC6785351

[53] Mason MJ, Schmidt C, Abraham A, Walker L, Tercyak K. Adolescents’ social environment and depression: social networks, extracurricular activity, and family relationship influences. J Clin Psychol Med Settings. 2009;16(4):346–54. doi: 10.1007/s10880-009-9169-4 19621251

[54] Kraus C, Kadriu B, Lanzenberger R, Zarate CA Jr., Kasper S. Prognosis and improved outcomes in major depression: a review. Transl Psychiatry. 2019;9(1):127. doi: 10.1038/s41398-019-0460-3 30944309 PMC6447556

[55] Tran KV, Esterman A, Saito Y, Brodaty H, Vu NC, Roughead E, et al. Factors Associated With High Rates of Depressive Symptomatology in Older People in Vietnam. Am J Geriatr Psychiatry. 2022;30(8):892–902. doi: 10.1016/j.jagp.2022.02.007 35339369

[56] Klaser K, Thompson EJ, Nguyen LH, Sudre CH, Antonelli M, Murray B, et al. Anxiety and depression symptoms after COVID-19 infection: results from the COVID Symptom Study app. J Neurol Neurosurg Psychiatry. 2021;92(12):1254–8. doi: 10.1136/jnnp-2021-327565 34583944 PMC8599635

[57] Levin AT, Hanage WP, Owusu-Boaitey N, Cochran KB, Walsh SP, Meyerowitz-Katz G. Assessing the age specificity of infection fatality rates for COVID-19: systematic review, meta-analysis, and public policy implications. Eur J Epidemiol. 2020;35(12):1123–38. doi: 10.1007/s10654-020-00698-1 33289900 PMC7721859

[58] Barrenetxea J, Pan A, Feng Q, Koh W-P. Factors associated with depression across age groups of older adults: The Singapore Chinese health study. Int J Geriatr Psychiatry. 2022;37(2):1–12. doi: 10.1002/gps.5666 34816486

[59] Kim J, Noh JW, Park J, Kwon YD. Body mass index and depressive symptoms in older adults: a cross-lagged panel analysis. PLoS One. 2014;9(12):e114891. doi: 10.1371/journal.pone.0114891 25501372 PMC4263712

[60] Tedders SH, Fokong KD, McKenzie LE, Wesley C, Yu L, Zhang J. Low cholesterol is associated with depression among US household population. J Affect Disord. 2011;135(1–3):115–21. doi: 10.1016/j.jad.2011.06.045 21802743

[61] Zhao L, Wang J, Deng H, Chen J, Ding D. Depressive Symptoms and ADL/IADL Disabilities Among Older Adults from Low-Income Families in Dalian, Liaoning. Clin Interv Aging. 2022;17:733–43. doi: 10.2147/CIA.S354654 35574289 PMC9091470

[62] Morin CM, Bjorvatn B, Chung F, Holzinger B, Partinen M, Penzel T, et al. Insomnia, anxiety, and depression during the COVID-19 pandemic: an international collaborative study. Sleep Med. 2021;87:38–45. doi: 10.1016/j.sleep.2021.07.035 34508986 PMC8425785

[63] Fang H, Tu S, Sheng J, Shao A. Depression in sleep disturbance: A review on a bidirectional relationship, mechanisms and treatment. J Cell Mol Med. 2019;23(4):2324–32. doi: 10.1111/jcmm.14170 30734486 PMC6433686

[64] Hackett ML, Pickles K. Part I: frequency of depression after stroke: an updated systematic review and meta-analysis of observational studies. Int J Stroke. 2014;9(8):1017–25. doi: 10.1111/ijs.12357 25117911

